# COVID-19 Seroprevalence in Romania: Insights from a Nationwide Antibody Study

**DOI:** 10.3390/epidemiologia6020026

**Published:** 2025-06-04

**Authors:** Réka Bodea, Toader Septimiu Voidăzan, Lorand Iozsef Ferencz, Zoltán Ábrám

**Affiliations:** 1 Department of Epidemiology, George Emil Palade University of Medicine, Pharmacy, Science, and Technology of Târgu Mureș, 540142 Târgu Mureș, Romania; reka.bodea@umfst.ro (R.B.);; 2 Department of Hygiene, George Emil Palade University of Medicine, Pharmacy, Science, and Technology of Târgu Mureș, 540142 Târgu Mureș, Romania

**Keywords:** seroepidemiological study, SARS-CoV-2, Immunoglobulin G, Romania, public health

## Abstract

Background: Frequency indicators are used by epidemiologists to facilitate public health professionals in estimating the impact of diseases. As of April 2022, Romania had reported 2.8 million confirmed COVID-19 cases to the World Health Organization, equating to a prevalence rate of 13.94%. A more accurate method for assessing the cumulative number of cases is the use of seroprevalence studies. This study retrospectively evaluates infection trends in Romania to enhance understanding of the virus’s spread and may support future comparative analyses of public health responses and community-level immunity. Methods: We analyzed 51,533 qualitative test results for high-affinity IgG antibodies targeting the SARS-CoV-2 nucleocapsid protein. Results: The largest proportion of tested individuals (59.4%) was aged 18–49 years. Among all serological tests, 18,980 were positive, corresponding to an adjusted seroprevalence rate of 40%. Conclusions: During the second year of the pandemic, seropositivity rates were highest among young adults, particularly in the western regions, and lowest among children and adolescents. These findings point out variations in exposure across age groups and geographic areas.

## 1. Introduction

Traditional epidemiological surveillance methods (calculation of frequency indicators, hospitalization rates, mortality tracking) have proven insufficient in capturing the full burden of the COVID-19 pandemic. While in the early stages of the pandemic, nucleic acid amplification tests, such as reverse-transcription polymerase chain reaction, were WHO-recommended confirmatory tests, additional research was required. The WHO guided researchers to population-based serological surveys to measure the disease’s magnitude and determine the surveillance system’s sensitivity. Seroprevalence studies can determine the percentage of the population with prior or late infection by detecting antibodies to SARS-CoV-2 in blood samples [1,2,3]. The body triggers a complex immune response against the virus when infected with SARS-CoV-2. SARS-CoV-2 is an RNA virus with structural proteins—spike (S), envelope (E), membrane (M), and nucleoprotein (N)—that play a key role in infection and replication [4]. The S protein enables viral entry, while the N protein is essential for genome packaging. After the innate immune response is triggered, the body recruits T cells to destroy infected cells and B cells to produce antibodies. SARS-CoV-2-specific neutralizing antibodies target viral entry mechanisms [5]. B cells stimulate the production of immunoglobulins (IgM, IgA, IgG) in response to viral antigens, such as structural proteins [6,7,8,9]. IgM is the first antibody to be produced in response to infection, and it can be detected as early as four days post-symptom onset (POS). Levels of IgM peak around day 20 before gradually declining [10]. IgG antibodies emerge after the initial IgM response and are detectable approximately 3 weeks POS [11,12]. While vaccines often target anti-S antibodies due to their association with neutralizing activity, not all anti-S antibodies necessarily exhibit neutralizing properties [13]. Antibodies against the N protein are commonly detected in individuals who have had a natural SARS-CoV-2 infection. Movsisyan et al. observed that IgG anti-N levels peaked around the fourth month post-infection, maintaining levels for the subsequent eight months, with a modest decline after that. They remained detectable by the end of the study (21 months) [14]. Grebe et al. highlight the utility of serologic detection of N antibodies and emphasize that they are critical tools for identifying previous SARS-CoV-2 infections, especially regarding spike-based vaccine uses [15]. Serological assays to detect IgG anti-N antibodies are commonly employed in epidemiological studies to estimate the prevalence of past SARS-CoV-2 infections in a population.

We note that in Romania, vaccines were used that produced anti-S antibodies: Pfizer-BioNTech (BNT162b2, Comirnaty), Moderna (mRNA-1273, Spikevax), AstraZeneca (ChAdOx1-S, Vaxzevria), and Johnson & Johnson (Ad26.COV2.S, Janssen), which supports our choice of laboratory test type [16]. Relatively few SARS-CoV-2 seroprevalence studies have been conducted in the country to assess the extent of infections. Most of these studies have focused on specific cohorts, such as urban populations, individual counties, or targeted groups (e.g., children, blood donors, and healthcare professionals). However, achieving comprehensive case ascertainment and reporting across a broader geographic area remains challenging [17,18,19,20].

This study’s primary objective is to retrospectively evaluate the seroprevalence of SARS-CoV-2 in the Romanian population during the 2020–2022 period. In addition, it explores potential disparities in exposure across different demographic and geographic subgroups.

## 2. Materials and Methods

### 2.1. Study Design

A retrospective cross-sectional study was conducted to assess the prevalence of SARS-CoV-2 antibody seropositivity.

### 2.2. Sample Collection and Study Population

A non-probability, convenience sampling method was employed. The sample consisted of participants who voluntarily sought SARS-CoV-2 serological testing for IgG anti-nucleocapsid (anti-N) antibodies at one of the MedLife Medical System laboratories across Romania. This testing was not part of a population-based screening program and, thus, is not expected to be a random sample of the Romanian population. Rather, it represents individuals with access to private healthcare services and a personal incentive to undergo testing due to prior symptoms, known exposure, occupational risk, or general health concerns. Romania, one of the states of Southeastern Central Europe, has a resident population of 19,053,815 people [21]. Between May 2020 and April 2022, covering the first two years of testing, a total of 51,533 blood samples were collected, analyzed, and interpreted. The laboratory submitted demographic information, such as age, gender, county of sample collection, and the number of times each individual had been tested. A total of 42,482 people were tested. Participants were stratified into different subgroups based on their ages and countries of origin. Age groups were categorized as 0–17 years, 18–49 years, 50–64 years, and ≥65 years. Age stratification was carried out based on the WHO guidelines as laid down in the Population-Based Age-Stratified Seroepidemiological Investigation Protocol for COVID-19 Virus Infection, March 2020 [22]. An unequal distribution of age groups was also identified within the sample. To improve accuracy and reduce potential bias, stratification was deemed necessary, as age can act as a confounding factor, potentially biasing the study results. Stratification of the data enables the study to take into account immunity–phenotype diversity in the population and to be more general.

Counties of origin were grouped according to the Nomenclature of Territorial Units for Statistics 2 (2024). Romania is divided into eight development regions, which (except București-Ilfov) are named according to their geographical position [23].

### 2.3. Laboratory Methods

Serological testing was performed using the Automated Abbott Accelerator platform (Manufacturer: Abbott Laboratories, City: Abbott Park, State: IL, Country: USA), based on the chemiluminescent microparticle immunoassay, for the qualitative detection of IgG anti-N antibodies against SARS-CoV-2. The following laboratory procedures were applied:

- Analyzer: ALINITY.
- Biological sample: Serum obtained by centrifugation at 3500 RPM for 10 min at 18 °C, processed on the same day or refrigerated for a maximum of 48 h post-collection.
- Cut-off value: 1.4 Index (Negative: <1.4, Positive: ≥1.4).
- Test performance: Specificity > 99.63%, Sensitivity 100% after the 14th day post-symptom onset.
- Regulatory approval: Certified for use in the European Union following Food and Drug Administration clearance in the United States [24,25].

### 2.4. Statistical Analysis

Statistical analyses were conducted using the Statistical Package for the Social Sciences (SPSS, version 23; Chicago, IL, USA). Quantitative variables, such as age, were presented as mean ± standard deviation (SD) for normally distributed data. Qualitative variables were expressed as counts and percentages. The Chi-square test was applied to compare prevalence rates between groups, considering the study design. All tests were interpreted in relation to statistical significance (*p* = 0.05), and statistical significance was considered for *p*-values under the significance threshold.

### 2.5. Ethical Considerations

This study was approved by the Ethics Committee of the George Emil Palade University of Medicine, Pharmacy, Science, and Technology of Târgu Mureș (3651/25 February 2025).

## 3. Results

### 3.1. Demographic Characteristics

**Figure 1:**
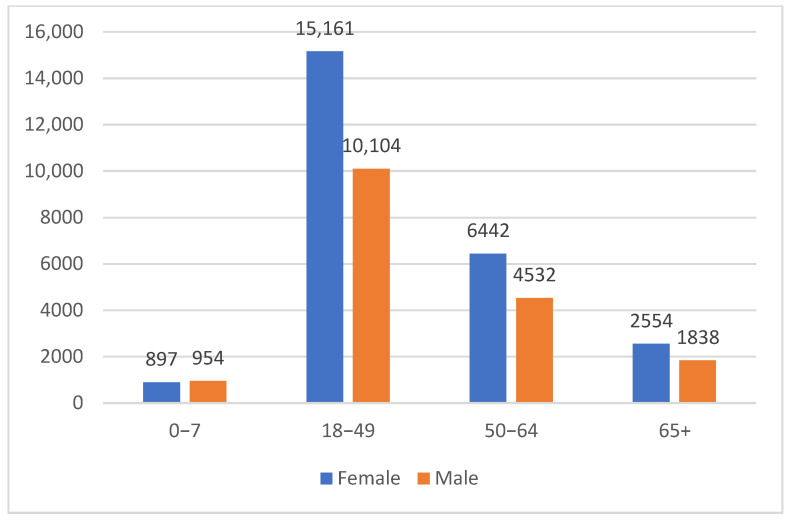
Characteristics of the study population—age and sex distribution.

Between May 2020 and April 2022, a total of 42,483 individuals visited one of the 34 MedLife laboratories in Romania and requested qualitative testing for SARS-CoV-2-specific IgG anti-N antibodies. The mean age of the participants was 44.87 ± 15.02 years (mean ± SD). The most represented age group was 18–49 years, comprising 59.4% of the study population (Figure 1).

**Figure 2:**
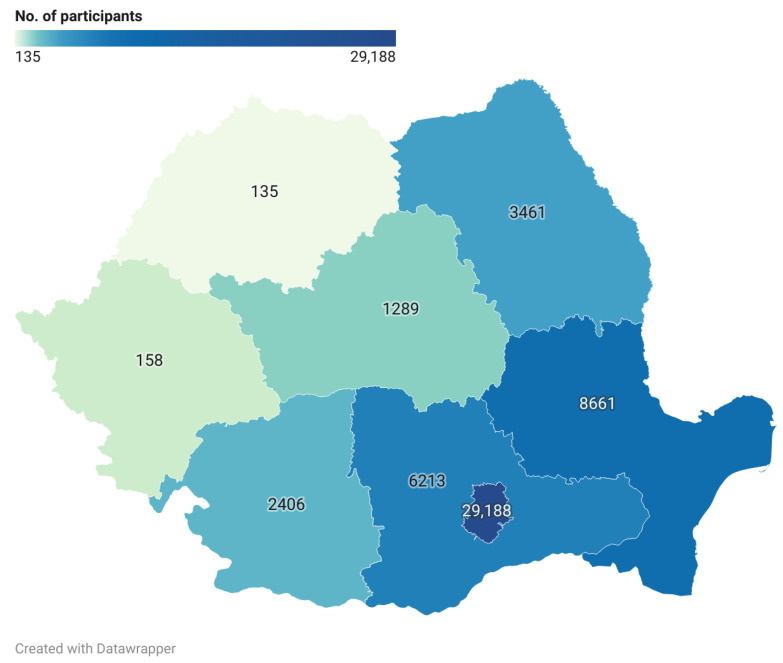
No. of performed serological tests divided by NUTS 2-level Romanian regions.

The majority of sample collections (56.6%) were conducted in the country’s capital, Bucharest. Notably, only 9.8% of Romania’s total population resides permanently in Bucharest (Figure 2).

The majority of tests (64.3%) were conducted in 2021, which was the only year tracked in its entirety. October 2021 recorded the highest number of tests, with 6200 (12%) conducted during that month.

### 3.2. Seroprevalence Results

**Figure 3:**
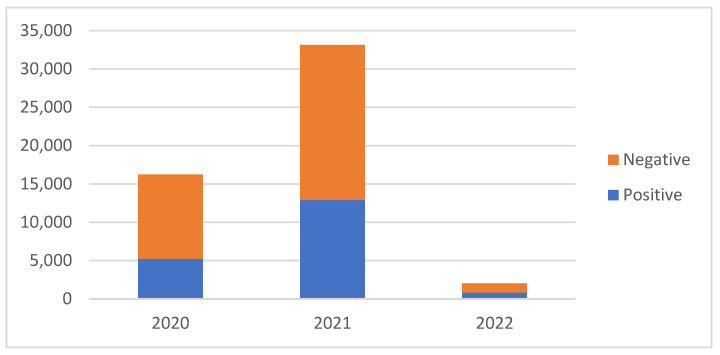
Seroprevalence results timeline.

According to the data, 18,980 serological tests were positive for SARS-CoV-2 IgG Anti-N antibodies. This represents a seroprevalence rate of 36.8%, indicating that more than one-third of individuals who underwent testing had been previously infected with SARS-CoV-2 [Figure 3].

A total of 5518 individuals were tested multiple times during the study period. Table 1 presents the number of test detections, along with the positive and negative results, as well as the corresponding seropositivity rate. After this adjustment, the total number of study participants is 42,482, resulting in a seropositivity rate of 40.02%.

It is important to note that the results presented below are based on all tests performed (n = 51,533). The age group with the highest IgG anti-N prevalence of seropositivity was adults aged 18–49 years (19.63%) (Table 2).

The highest seropositivity rate was observed in December 2021, with 60% of the tests performed yielding positive results. Regional variations in seroprevalence are presented in Table 3, with the Central region exhibiting the highest rate at 46.3% (Table 3). The percentage of positive cases by region over time is shown in Table 4. In the Southwest region, the positivity rate reached 63% in 2022. The analysis of participants by age category, region, and seropositivity is summarized in Table 5. In all eight macroregions of the country, young adults had the highest seropositivity rates (ranging from 1.78% to 69.62%). Additionally, the western part of the country displayed the highest seropositivity rates across all age categories (Table 5).

## 4. Discussion

The study aimed to determine the seroprevalence rate of SARS-CoV-2 IgG anti-N antibodies in the Romanian population who self-reported for testing during the first two years of the COVID-19 pandemic, with the goal of understanding population-level exposure and behavioral patterns in response to the COVID-10 pandemic. By examining the distribution of SARS-CoV-2 antibodies, the research is offering a contextual reference point for evaluating the effectiveness of public health communication and public adherence to guidelines. Although not intended to inform current policy decisions, the findings contribute to the historical record of pandemic dynamics and may support future comparative analyses of public health responses and community-level immunity.

Romania reported its first cases at the western border in late February 2020 [26]. In response to the increasing number of infections, a state of emergency was declared on 16 March 2020. On 25 March, additional restrictions were implemented through a military ordinance [27]. Schools were closed on 11 March 2020, and remained closed until September 2020. Eligibility for vaccination for the general adult population began on 15 March 2021 [18]. The national vaccination campaign officially started on 27 December 2020, with the first vaccine administered to a healthcare and social worker from both the public and private sectors. This marked the beginning of Romania’s COVID-19 vaccination strategy. In the second phase of the campaign, high-risk individuals and essential sector workers were prioritized for mass immunization. On 15 March 2021, eligibility was extended to the general adult and pediatric populations of part of the third phase [28].

By April 2022, the WHO reported 2.8 million confirmed COVID-19 cases in Romania [29]. The disease prevalence was 4.08% in the first year and increased to 13.94% in the second year [30]. A 2022 study by Enciu et al. compared Romania with neighboring countries, finding that Romania ranked fourth for cumulative case incidence in Europe [31]. Between February 2020 and February 2022, Romania experienced the circulation of several SARS-CoV-2 variants, each influencing the course of the COVID-19 pandemic in the country. During the early stages of the pandemic (February 2020–Early 2021), the original SARS-CoV-2 strain predominated in Romania, characterized by its initial spread and the onset of the first wave of infections. Subsequently, the Alpha variant (B.1.1.7) emerged in late 2020 and remained prevalent through early 2021. By mid-2021, the Delta variant (B.1.617.2) became the dominant strain, notably contributing to the third wave of infections. By early 2022, Omicron (B.1.1.529) and its sub-linages began to circulate in Romania. These variants were characterized by a high number of mutations in the spike protein and were associated with increased transmissibility [32,33]. Throughout this period, Romania implemented a range of public health measures to mitigate the spread of these variants, including lockdowns, vaccination campaigns, and travel restrictions. Changes in public health policies, along with shifts in individual behaviors, may have also contributed to increased transmission and a subsequent rise in confirmed case numbers.

We would like to draw attention to the substantial methodological differences among seroprevalence studies, making direct comparisons challenging them, and limiting the discussions of our findings to them. Although we aimed to discuss studies published between 2020 and 2022, variations in sampling methods, data collection, and the type of antibodies tested (indicated in parentheses) further complicate valid comparisons. In the next Section, we discuss seroprevalence studies conducted in Romania and neighboring Eastern European countries.

The first published Romanian seroprevalence survey, in which anti-SARS-CoV-2 IgG antibodies by chemiluminescent technology were detected, concluded a 6.19% rate (95% CI: 5.85–6.53) between June and October 2020, suggesting infections were five times higher than reported cases [17]. Our study, using the same convenience sampling method but with a smaller sample (9146), found an IgG anti-N seroprevalence rate of 22.97% for the same period. In Region B, the seroprevalence rate (25.37%) exceeded the 3.55% reported in the Pistol et al. study, which had a low enrollment rate in that region. Most positive cases were in young adults (18–49 years), likely due to increased testing for professional reasons.

A Serbian study in Vojvodina observed a seroprevalence (by detecting IgG anti-S and anti-N) increase from 2.60% to 16.67% between April and September 2020, indicating widespread underreporting of cases [34]. Another Serbian study (June–October 2020) found an 18.3% seroprevalence (by detecting IgG anti-S and anti-N) among healthcare workers, peaking at 28.6% in COVID-designated hospitals [35].

In Hungary, a study between April 2020 and March 2021 estimated a seroprevalence (by detecting IgM, IgA, IgG anti-S and anti-N) of 3.3–4.9%, implying early SARS-CoV-2 circulation in Budapest [36].

During Romania’s fourth COVID-19 wave (October 2021), daily cases neared 20,000, with over 500 deaths per day, straining the healthcare system [37,38]. Based on our results in October 2021, we recorded the highest number of tests (6200, respectively 12% of the total). A study conducted in December 2021 in western Romania found a 45.60% seroprevalence (by detecting IgM, IgA, and IgG anti-N) among blood donors—six times higher than confirmed cases. Another study by Olariu et al. (April 2022) in Timiș County found a 46.70% seroprevalence in children, similar to adults in the same period, while our nationwide seropositivity among children was 1.67% [18,20]. The seropositivity rates in our study among children ranged from 1.21% to 3.16%, suggesting that children’s contribution to the asymptomatic transmission of SARS-CoV-2 in the community was likely small.

International studies suggest that younger children have lower SARS-CoV-2 antibody prevalence, possibly due to school closures or reduced susceptibility [39]. A September 2022 meta-analysis found children were not primary contributors to household transmission, though their transmissibility increased with emerging variants [40]. Naeimi et al. reported that, by late 2021—before the Omicron wave—50–70% of children remained susceptible to infection. Seroprevalence was higher in disadvantaged regions, ethnic minorities, and older children, rising from 7.3% (95% CI: 5.8–9.1%) in the first pandemic wave to 37.6% (95% CI: 18.1–59.4%) in the fifth and 56.6% (95% CI: 52.8–60.5%) in the sixth [41]. Nantel et al., in a preprint study, point out that antibody responses were similar between pediatric and adult populations; also, children had a weaker cellular (T-cell) immune memory to SARS-CoV-2 than adults. They suggest a key difference in long-term immunity mechanisms between age groups [42].

After discussing several studies from the Eastern European region, we need to mention seroprevalence studies that summarize global data, analyzing the same period as our study. Seroprevalence surveys conducted in the United States and Europe between 2020 and 2022 suggest that, after adjusting for potential false positives or negatives, the true rate of SARS-CoV-2 exposure exceeds the reported incidence of cases by approximately 10-fold or more [43,44,45,46]. In November 2022, Bergeri et al. published a systematic review and meta-analysis on the global seroprevalence of SARS-CoV-2, covering studies conducted between January 2020 and April 2022. This meta-analysis, which standardized the results according to the WHO Unity Protocol, estimated the extent of population infection and seropositivity over the two-year pandemic period. By September 2021, the global seroprevalence of SARS-CoV-2, from both infection and vaccination, was 59.2% [47].

We underscore the importance of conducting seroprevalence surveys to more accurately estimate the burden of communicable diseases. Although the studies employ different sampling and laboratory testing methods, they all reveal a higher number of infections than initially reported, allowing for a more precise estimation of the infectious risk posed by the virus. In our study, individuals aged 18–49 years constituted the majority of those tested (see Table 3), representing the most active segment of the population with frequent interactions across age groups, including both children and the elderly. This age group is also more likely to engage in health-seeking behaviors and fulfill occupational responsibilities that may require travel or testing due to workplace exposure. Notably, despite their high testing rates, the 18–49 age group accounted for the lowest proportion of positive cases. This finding may reflect a higher level of compliance with preventive measures and a heightened sense of personal and community responsibility within this demographic. Women were also overrepresented, and children (ages 0–17 years) comprised only 3.75% of the sample. Additionally, there were significant regional discrepancies, with B representing 56% of the sample, while the NW region accounted for only 0.35%.

McConnel et al. spotlight the importance and challenges of COVID-19 seroprevalence studies [48]. Differences in demographics, behaviors, and risk factors may skew findings. Since testing entire populations is impractical, seroprevalence studies rely on sampling, where accuracy depends on representativeness. Selection bias, such as overrepresentation of HCWs or blood donors, can distort results [48]. Test accuracy is essential in epidemiological studies. Seroprevalence studies use antibodies as infection markers, but waning immunity and resistant variants complicate estimates [48]. Serological assays targeting the N protein are widely used to assess previous SARS-CoV-2 infection; however, growing evidence suggests variability in the duration of the antibody response. While certain studies indicate the persistence of anti-N antibodies [14,15], others demonstrate that these antibodies may diminish to undetectable levels within months after primary infection [49,50,51,52]. This discrepancy may have contributed to the lower seroprevalence observed in 2022 compared to 2021, potentially leading to an underestimation of the cumulative infection rate. Another possible explanation may lie in temporal changes in testing behavior, including decreased public interest in serological testing during the later stages of the pandemic and shifts in the demographic or occupational profiles of individuals who underwent testing, which may have influenced the observed seroprevalence trends. On the other hand, a key strength of our study is the use of the IgG anti-N test with high specificity (>99.63%) and sensitivity (100%) after the 14th day post-symptom onset. The large sample size (51,533 performed tests from 42,482 persons) further enhances the reliability of our results.

Future studies should aim to employ population-representative sampling methods to generate more generalizable estimates of SARS-CoV-2 seroprevalence in Romania. Longitudinal research incorporating both humoral and cellular immune responses is warranted to assess the duration and quality of immunity, particularly among children and other underrepresented groups.

### Study Limitation

This study has limitations that should be considered when interpreting the results. First, the results are not generalizable due to the non-probability, convenience sampling strategy. Selection bias may be introduced by the fact that the study population consisted of self-selected individuals who volunteered for antibody testing at private laboratories. These persons likely differ from the general population in terms of health-seeking behavior, perceived exposure, or access to medical services. Second, the lack of clinical and epidemiological data (symptom history, prior PCR confirmation, or vaccination status) restricts the ability to distinguish between infection- and vaccine-induced immunity. Lastly, the study exclusively quantified IgG antibodies targeting the nucleocapsid (N) protein. Moreover, sample distribution in the regions was unbalanced, with an evident overrepresentation from Bucharest and underrepresentation from other regions, such as the North-West, which may have affected the regional comparisons. Finally, as this is a retrospective, cross-sectional study, the analysis is inherently limited in tracking temporal variations in individual immune responses and in establishing causal relationships.

## 5. Conclusions

This retrospective, cross-sectional study showed that the seroprevalence of SARS-CoV-2 in Romania reached nearly 40% by April 2022, which is much higher than the officially reported case numbers for the same period. The highest seropositivity rates were recorded among young adults (18–49 years old), especially in the western regions, and the lowest among children and adolescents. These results highlight differences in exposure between age groups and geographical areas.

## Figures and Tables

**Table 1:** The seroprevalence rate by number of detections.

		SARS-CoV-2 Antibody Test Results No.		
No. of Detectations	No. of Participants (42,482)	Negative (25,478)	Positive (17,004)	Prevalence of Seropositivity (40.02%)
1	36,964	22,867	14,097	38.13%
2	3693	1704	1989	53.85%
3	957	459	498	52.03%
4	433	234	199	45.95%
5	237	132	105	44.30%
6	107	46	61	57.00%
7	37	13	24	64.86%
8	29	13	16	55.17%
9	11	6	5	45.45%
10	7	1	6	85.71%
11	3	1	2	66.66%
14	2	0	2	100%
16	2	2	0	0%

**Table 2:** The seroprevalence rate by age group.

Age	Prevalence of Seropositivity
0–17	1.67%
18–49	19.63%
50–64	10.74%
65+	4.81%

**Table 3:** Seroprevalence results in multivariate analysis.

Variable	SARS-CoV-2 Antibody Test Results No. (%)			*p* Value
	**Negative** 32,553 (63.2%)	**Positive** 18,980 (36.8%)	**Total** 51,533	
Sex				0.001
Female	19,568 (63.8%)	11,121 (36.2%)	30,689	
Male	12,985 (62.2%)	7859 (37.8%)	20,844	
Age (yrs)				0.0001
0–17	1077 (55.6%)	859 (44.4%)	1936	
18–49	21,059 (67.5%)	10,114 (32.5%)	31,173	
50–64	7853 (58.7%)	5528 (41.3%)	13,381	
65+	2564 (50.8%)	2479 (49.2%)	5043	
Testing period (Year)				0.0001
2020	11,009 (67.8%)	5226 (32.2%)	16,235	
2021	20,253 (61.1%)	12,905 (38.9%)	33,158	
2022	1212 (60.0%)	809 (40.0%)	2021	
Regions				0.0001
Bucharest	18,761 (64.3%)	10,427 (35.7%)	29,188	
Central	692 (53.7%)	597 (46.3%)	1289	
North-East	2200 (63.5%)	1261 (36.5%)	3461	
North-West	131 (97.0%)	4 (3.0%)	135	
South	3550 (57.1%)	2663 (42.9%)	6213	
South-East	5567 (64.2%)	3094 (35.8%)	8661	
South-West	1510 (62.7%)	896 (37.3%)	2406	
West	131 (82.9%)	27 (17.1%)	158	

**Table 4:** % of seropositive cases by regions and years. Regions: B—Bucharest, C—Center, NE—North-East, NW—North-West, S—South, SE—South-East, SW—South-West, W—West.

Region	Year		
	2020	2021	2022
B	34%	36%	38%
C	40%	46%	43%
NE	28%	43%	38%
NW	3%	ND	ND
S	39%	45%	40%
SE	25%	40%	43%
SW	33%	37%	63%
W	17%	0%	ND

**Table 5:** The seroprevalence rate by age group and region. Regions: B—Bucharest, C—Center, NE—North-East, NW—North-West, S—South, SE—South-East, SW—South-West, W—West.

Age	Region							
	B	C	NE	NW	S	SE	SW	W
0–17	1.74%	1.4%	1.21%	0%	1.43%	1.56%	2.62%	3.16%
18–49	20.11%	25.68%	19.36%	1.48%	21.63%	16.45%	18.54%	69.62%
50–64	9.64%	13.73%	11.1%	0.74%	13.5%	12.19%	10.47%	19.62%
65+	4.23%	5.51%	4.74%	0.74%	6.29%	5.52%	5.61%	7.59%

## Data Availability

The data presented in this study are not publicly available due to privacy and ethical restrictions.

## Abbreviations

The following abbreviations are used in this manuscript:

S	Spike
E	Envelope
M	Membrane
N	Nucleocapsid
POS	post-symptom onset
CMIA	chemiluminescent microparticle immunoassay
B	Bucharest
C	Center
NE	North-East
NW	North-West
S	South
SE	South-East
SW	South-West
W	West
HCW	healthcare workers
SARS-CoV-2	Severe Acute Respiratory Syndrome Coronavirus-2
WHO	World Health Organization
COVID-19	Coronavirus Disease 2019
